# Irritability in preschool children with attention‐deficit/hyperactivity disorder: Analysis of family environmental factors

**DOI:** 10.1002/jcv2.70120

**Published:** 2026-04-08

**Authors:** Analin Ono Baraniuk, Giovanni Abrahão Salum, David Daley, Erika Mendonça de Morais, Filipe Jaeger Zabala, Luiz Eduardo de Souza, Guilherme V. Polanczyk, Luisa Shiguemi Sugaya

**Affiliations:** ^1^ Division of Child and Adolescent Psychiatry, Department & Institute of Psychiatry Hospital das Clínicas Faculty of Medicine University of São Paulo (FMUSP) São Paulo Brazil; ^2^ National Institute of Developmental Psychiatry CNPq São Paulo Brazil; ^3^ Child Mind Institute New York New York USA; ^4^ Department of Psychiatry Hospital de Clínicas de Porto Alegre (HCPA) Federal University of Rio Grande do Sul (UFRGS) Porto Alegre Brazil; ^5^ NTU Psychology School of Social Sciences Nottingham Trent University Nottingham UK; ^6^ Institute of Psychiatry Faculty of Medicine University of São Paulo (FMUSP) São Paulo Brazil; ^7^ Pontifical Catholic University of Rio Grande do Sul (PUCRS) Porto Alegre Brazil; ^8^ Data Science Pontifical Catholic University of Rio Grande do Sul (PUCRS) Porto Alegre Brazil

**Keywords:** attention‐deficit/hyperactivity disorder, family environmental factors, irritability, maternal psychopathology, parenting, preschoolers

## Abstract

**Background:**

Irritability affects one‐third of children and adolescents with attention‐deficit/hyperactivity disorder (ADHD) and is associated with negative outcomes. The family environment plays a prominent role in the child's development, and therefore on the risk for irritability, especially during the preschool period. The aim of this study was to investigate the association between multiple family environmental factors and irritability in preschool children with ADHD.

**Methods:**

This is a cross‐sectional analysis nested within a randomized clinical trial with preschool children with ADHD—the MAPPA study. Here, 128 children (107 males, 21 females) aged 3–5 years with complete data were included. Irritability was measured by the Affective Reactivity Index. Three dimensions of family environmental factors were considered: parenting, family environment, and maternal characteristics (comprising maternal psychopathology, temperament, and cognitive ability). Analyses were conducted to evaluate the association of individual factors with irritability, followed by a multiple linear regression model.

**Results:**

Associations were found between irritability and both parenting and maternal characteristics, particularly psychopathology and temperament. The multiple linear regression model revealed a greater frequency of negative over positive comments in the Preschool Five‐Minute Speech Sample alongside less positive parenting practices and maternal ADHD symptoms as the family environmental factors significantly associated with child irritability. Findings remained significant after controlling for child sex and child ADHD symptom severity.

**Conclusion:**

Our findings indicated that maternal individual characteristics and parenting practices are associated with irritability in preschool children with ADHD, supporting the importance of family environment in understanding this condition. A thorough assessment of parenting practices and parental psychopathology is relevant when planning interventions aimed at reducing irritability in preschoolers with ADHD.

## Key Points

**What's Known?**

- Both irritability and difficulties within the family environment are common in children with attention‐deficit/hyperactivity disorder (ADHD), but their effect over irritability during the preschool years remains underexplored.

**What's New?**

- This is the first study to evaluate the association between various family environmental factors and irritability in preschool children with ADHD.
- Results showed that parenting and maternal psychopathology played an important role. Specifically, a higher frequency of negative over positive comments in the Preschool Five‐Minute Speech Sample, less positive parenting practices and maternal ADHD symptoms emerged as the factors associated with child irritability.

**What's Relevant?**

- Our study advances the understanding of key family environmental factors related to irritability in preschoolers with ADHD, suggesting potential targets for family‐based early intervention.

## INTRODUCTION

Irritability is defined as proneness to anger that can be developmentally expected but may be impairing and becomes clinically relevant when it occurs frequently, is not appropriate to the child's age and disproportionate to the situation (Leibenluft et al., 2024). It is one of the leading reasons for mental health referrals in childhood and is a transdiagnostic construct shared across multiple disorders, including depression, oppositional defiant disorder (ODD) and attention‐deficit/hyperactivity disorder (ADHD) (Evans et al., 2024; Shakeshaft et al., 2026).

In individuals with ADHD, irritability is highly prevalent, with approximately one‐third of children and adolescents experiencing clinically significant levels (Breaux et al., 2022). Irritability is closely linked to ADHD symptom severity and is related to both internalizing and externalizing problems (Galera et al., 2021; Silva Santos et al., 2025). Longitudinal studies indicate that chronic irritability in children with ADHD is associated with several negative outcomes later in life, such as depression, anxiety, and a higher risk of suicide (Eyre et al., 2019; Galera et al., 2021; Sorcher et al., 2022).

Although the literature suggests a genetic influence, with an association between early irritability and polygenic risk scores for ADHD (Nigg et al., 2020; Riglin et al., 2017, 2019), environmental factors such as negative parenting also play a role (Oliver, 2015). The family environment represents the first social setting, and through interactions within the family children develop competencies and skills, including emotion regulation (Claussen et al., 2022; Zeman et al., 2006). Inconsistent parenting practices and reduced expressions of affection and empathy have been correlated with irritability in childhood (Brotman et al., 2017; Strayer et al., 2004). Also, parents who are more reactive and irritable may struggle to provide sufficient emotional and behavioral support to help their children develop appropriate self‐regulation (Gershy & Gray, 2020).

Parents of children with ADHD often experience higher levels of stress compared to families with typically developing children (Breaux & Harvey, 2019; Haneda et al., 2025), which may intensify caregiving difficulties. The maternal caregiving context warrants special attention, as mothers are most often the primary caregivers during early childhood and the main informants in both clinical and research settings. Mothers of children with ADHD are more likely to exhibit invalidating responses and often report a lower sense of parental competence, defined as perceived efficacy and satisfaction in the parenting role (Harrison & Sofronoff, 2002; Jones et al., 2025). Additionally, they frequently demonstrate lower involvement with their children, reduced marital satisfaction, and increased family conflicts, which can contribute to an unfavorable family environment (Johnston & Mash, 2001). Parental psychopathology is also more prevalent in these families (Breaux & Harvey, 2019; Harrison & Sofronoff, 2002), with maternal depression emerging as a significant predictor of behavioral and emotional problems in offspring (Breaux & Harvey, 2019; Mulraney et al., 2017).

The investigation of the relationship between irritability and difficulties within the family environment during the preschool years is particularly valuable. This period is critical for the establishment of emotional and behavioral patterns, and the quality of the parent‐child relationship at this stage plays a key role in long‐term development and may be more amenable to change. Different specific dimensions of family environment are correlated between them and may confound specific associations with irritability. Frequently, studies evaluate only a few dimensions or aspects of caregiver behavior and psychopathology (Ravi et al., 2023; Zendarski et al., 2023), thus providing an incomplete and confounded picture. No previous study has examined different family environment components to identify which factors are independently associated with irritability in preschoolers with ADHD. A deeper understanding of this topic could inform target interventions, particularly considering the scarce literature on non‐pharmacological approaches aimed at reducing irritability in children with ADHD (Breaux et al., 2022).

The MAPPA study was a randomized clinical trial primarily designed to evaluate the efficacy of methylphenidate and behavioral parenting training in children aged 3–5 years with ADHD. Primary results showed that methylphenidate was effective in reducing ADHD symptoms and improving functionality, and behavioral parent training was effective in improving functionality for preschool children with ADHD after 8 weeks of treatment. Notably, the trial also highlighted that irritability is frequent in this population, and revealed that behavioral parent training was more effective for reducing irritability than methylphenidate or placebo combined with psychoeducational intervention (Sugaya, Salum, et al., 2022). This finding suggests that family‐based interventions may play a central role in the management of irritability in preschoolers with ADHD. However, it remains unclear which specific family environmental factors are associated with irritability and may represent key targets for therapeutic approach. Therefore, the present study sought to explore whether multiple components—such as family dysfunction, negative parenting behaviors (e.g., criticism and hostility), household chaos, and maternal characteristics, including psychopathology—are associated with higher irritability in these children.

## METHODS

### Study design and participants

A cross‐sectional observational analysis was conducted as a nested sub‐study within the MAPPA study, an 8‐week, randomized, double‐blind, placebo‐ and sham parenting training‐controlled clinical trial in children aged 3–5 years with moderate‐to‐severe ADHD held at the Institute of Psychiatry, Hospital das Clínicas, University of São Paulo Medical School (IPq‐HC‐FMUSP), Brazil. It investigated the efficacy and safety of methylphenidate and behavioral parent training in reducing the frequency and severity of ADHD symptoms and improving global functioning (Sugaya, Salum, et al., 2022). This trial was completed and registered with ClinicalTrials.gov—NCT02807870.

The MAPPA study included 153 children diagnosed with ADHD according to the Diagnostic and Statistical Manual of Mental Disorders, 5th Edition (DSM‐5) (APA, 2013). Inclusion criteria were score above 32 on the Swanson, Nolan, and Pelham‐IV (SNAP‐IV) scale (indicating clinically significant ADHD symptoms), and being registered in preschool or daycare. Clinical assessment and diagnosis were conducted by six child and adolescent psychiatrists. Diagnosis was based on the semi‐structured interview Kiddie Schedule for Affective Disorders and Schizophrenia Lifetime Version (K‐SADS‐PL) (Caye et al., 2017) following the Research Diagnostic Criteria–Preschool Age (Scheeringa et al., 2003), an adaptation for this age group. The exclusion criteria were: intelligence quotient (IQ) < 70; diagnosis of affective disorders, psychotic disorders, or autism spectrum disorder; a major clinical condition or history of neurological disorder or head trauma; the use of psychotropic medications during the previous 30 days; and the absence of a legal guardian capable of understanding the study's objectives and instructions. The parent or primary caregiver of each participant provided a written informed consent.

According to the MAPPA study protocol, only mothers recorded the Preschool Five‐Minute Speech Sample (PFMSS), given that mothers were expected to be the primary caregivers. Of the 153 individuals, 144 mothers completed the PFMSS. Here, only the pre‐intervention data of children with complete datasets (*n* = 128) were included in the final analytical sample. The final sample had a mean age of 5.0 years (SD = 0.6), and was predominantly male (*n* = 107, 83.6%). A description of the sociodemographic and clinical characteristics is provided in Table 1.

**TABLE 1:** Descriptive characteristics of the sample.

Variable	Value
Sex, *n* (%)	
Male	107 (83.6%)
Female	21 (16.4%)
Age in months, *M* (SD)	60.89 (7.4)
Family monthly income, *M* (SD), US$	1283.03 (1365.59)
IQ score caregiver, *M* (SD)	103.19 (14.88)
ADHD presentation, *n* (%)	
Inattention	9 (7.0%)
Hyperactivity/impulsivity	28 (21.9%)
Combined	91 (71.1%)
Comorbidities, *n* (%)	
Oppositional defiant disorder	53 (41.4%)
Anxiety disorders	35 (27.3%)
Enuresis or encopresis	15 (11.7%)
Other disorders	23 (18.0%)
ARI, *M* (SD); median (IQR)	5.99 (3.07); 6 (3–8)
SNAP‐IV, *M* (SD)	
Total	35.16 (8.84)
Inattention	16.54 (4.89)
Hyperactivity/impulsivity	18.62 (5.01)

Abbreviations: ADHD, attention‐deficit/hyperactivity disorder; ARI, Affective Reactivity Index; IQ, intelligence quotient; IQR, interquartile range; *M*, mean; SD, standard deviation; SNAP‐IV, Swanson, Nolan, and Pelham‐IV scale; US$, United States dollar.

### Measures

Each measure of interest was focused on one of the following dimensions: child irritability, parenting, family environment, and maternal characteristics.

#### Child irritability

The child's irritability was measured using the Brazilian Portuguese version of the Affective Reactivity Index (ARI) (DeSousa et al., 2013). This 7‐item parent‐ and self‐report instrument has strong psychometric properties (Stringaris et al., 2012) and has demonstrated good validity and reliability in preschoolers (Sugaya, Kircanski, et al., 2022; Wilson et al., 2022). The ARI covers the threshold for an anger reaction, as well as the frequency and duration of children's irritability. For the first six items, responses are on a three‐point Likert scale, scored from 0 = not true to 2 = certainly true. The total score is the sum of the six items, ranging from 0 to 12, with higher scores indicating greater levels of irritability. The seventh item indicates level of impairment. Here, we used the parent‐report version, and the internal consistency was good (Cronbach's *α* = .87).

#### Parenting

Each measure of parenting is described below.

The Parenting Scale (PS; Arnold et al., 1993) is a 28‐item measure of dysfunctional discipline practices across three subscales: laxness (permissive discipline), over‐reactivity (exaggerated parental reactions to the child's behavior), and hostility. Each item is constructed as a hypothetical situation (e.g., When my child misbehaves…), with two possible parental responses (e.g., “I raise my voice or yell” and “I speak to my child calmly”). Using a 7‐point Likert scale, parents are asked to choose which behavior best describes their parenting style. In our study, the total score—the average of responses across all items—was considered, with higher scores indicating more dysfunctional practices.

The Parenting Sense of Competence Scale (PSOC; Johnston & Mash, 1989) is a 17‐item instrument that assesses a parent's perceived competence, encompassing two subscales that evaluate both self‐efficacy and satisfaction in their parenting role. Respondents rate each statement on a 6‐point Likert scale, ranging from “Strongly agree” to “Strongly disagree.” Here, the total scale score was used, with higher scores reflecting a stronger sense of parental competence.

The Parent Practices Scale (PPS; Strayhorn & Weidman, 1988) is a 34‐item measure that examines how parents interact with their children and investigates their parenting practices. Each item is rated on a 6‐point Likert scale, ranging from 1 = never to 6 = many times a day. In this study, the total score was considered, with higher scores representing more positive and supportive parenting practices, such as reinforcement of adaptive behaviors, clear limit‐setting, warmth, and emotional support.

The PFMSS (Daley et al., 2003) is a valid and reliable instrument for assessing Expressed Emotion (EE) in parents of young children. EE is a qualitative measure that evaluates the attitudes and feelings that individuals express about their family members, characterizing parenting behaviors and the quality of parent‐child interactions. High EE is associated with negative parenting behaviors (Cartwright et al., 2011) and refers to the display of hostility, criticism, and emotional overinvolvement (overprotection and self‐sacrifice) (Magaña‐Amato, 1989; Smith et al., 2022). Increased levels of criticism identified by EE are commonly observed in the relationship between caregivers and children with psychiatric disorders (Psychogiou et al., 2017), including ADHD (Cartwright et al., 2011). In the PFMSS, parents describe their child and their relationship with them through a 5‐min recording speech, without interruption (Daley et al., 2003). In our study, the mothers' records were transcribed and coded by a trained rater. Beyond EE, additional and independent dimensions relevant to the preschool stage were examined (Daley et al., 2003). These dimensions are listed in Table 2.

**TABLE 2:** Domains included in the FMSS version for preschoolers (PFMSS).

Domains of PFMSS	Description	Response categories	Example
Initial statement	First thought expressed by the mother	Positive	“Luke is a very loved boy.”
		Negative	
		Neutral	
Warmth	Intensity of the feeling that the mother expresses about the child, based on the tone of voice, spontaneity, concern, and empathy	High	“I love my son and I am blessed to have him in my life.”
		Moderate	
		Low	
Relationship	Quality of the relationship and activities between mother and child, based on the report about the relationship and the mother's expression of liking and valuing the time spent with the child	Positive	“My son and I have a very good relationship.”
		Negative	
		Neutral	
Frequency count of critical comments	Statements that criticize the child	Count of comments	“He is very aggressive and does not obey me.”
Frequency count of positive comments	Statements of praise or appreciation for the child	Count of comments	“He is a very good boy.”

*Note*: Adapted from Daley et al. (2003).

Abbreviation: FMSS, Five‐Minute Speech Sample; PFMSS, Preschool Five‐Minute Speech Sample.

#### Family environment

The Confusion, Hubbub, and Order Scale (CHAOS; Matheny et al., 1995) is a 15‐item instrument that investigates the daily home atmosphere. Seven items assess routine and organization, while eight reflect disorganization, confusion, and noise. Caregivers indicate whether each statement is true (1 point) or false (0 points). Higher total scores reflect greater levels of chaos and disorganization within the household.

#### Maternal characteristics

Maternal characteristics were subdivided into psychopathology, temperament and cognitive ability. Each measure is described below.

##### Maternal psychopathology

The Beck Depression Inventory‐II (BDI‐II; Beck et al., 1996) is a 21‐item measure that examines depressive symptoms in adults, with established validity and reliability in the Portuguese language (Gomes‐Oliveira et al., 2012). The items are based on the criteria for major depression (DSM‐IV; APA, 1994), and are scored from 0 to 3, yielding a total score from 0 to 63. Higher scores indicate greater severity of depression. In our study, the BDI‐II was treated as a continuous variable, using the total score for analysis.

The Adult Self‐Report Scale (ASRS; Kessler et al., 2005) is an 18‐item questionnaire designed to assess ADHD symptoms in adults, covering inattention and hyperactivity/impulsivity domains in accordance with DSM‐IV‐Text Revision criteria for this condition (APA, 2000). Respondents rate each item on a 5‐point scale indicating symptom frequency, from 0 = never to 4 = very often. In our study, the ASRS was treated as a continuous variable, with the total score considered in the analysis.

##### Maternal temperament

The Adult Temperament Questionnaire (ATQ; Evans & Rothbart, 2007) is a 77‐item instrument that assesses the primary caregiver's temperament. Each item is rated on a 7‐point Likert scale ranging from 1 = extremely false to 7 = extremely true. The ATQ evaluates four dimensions of temperament: negative affect (heightened sensitivity to negative stimuli), effortful control (ability to regulate attention and inhibit impulses), extraversion (sociability, enjoyment of social interactions and high activity levels), and orienting sensitivity (perception of subtle environmental cues). In our study, the scores of each dimension were included in the analysis.

##### Maternal cognitive ability

The Wechsler Adult Intelligence Scale‐III (WAIS‐III; Wechsler, 1997) is a standardized instrument designed to assess intellectual capacity and overall cognitive functioning of adults and older adolescents. It comprises multiple subtests covering domains such as verbal comprehension, perceptual organization, working memory, and processing speed, which are combined to yield the Full Scale IQ. In our study, the analysis was based only on the Full Scale IQ score.

#### Covariates

Child sex and ADHD symptom severity were included as covariates, given the marked male predominance in the sample and the well‐established association between irritability and ADHD symptom severity (Galera et al., 2021). Child ADHD symptoms were evaluated using the SNAP‐IV (Swanson et al., 2001), a 26‐item scale that assesses symptoms of ADHD and ODD, rated on a 4‐point Likert scale (0 = not at all to 3 = very much). The first 18 items are used to compute inattention (range 0–27), hyperactivity/impulsivity (range 0–27), and total ADHD (range 0–54) scores. Here, parent ratings at baseline were used.

### Statistical analysis

Data were collected via REDCap (Harris et al., 2009, 2019) and analyzed in R 4.3.1/RStudio 2023.6.1.524. We initially examined the association of each variable with ARI, treated as a continuous variable. Non‐parametric tests were applied in the exploratory analyses because ARI total scores deviated from normality. To ensure consistency across analyses, Spearman correlations were employed for continuous variables, and the Wilcoxon test was used to compare ARI scores by sex. For the qualitative PFMSS, median ARI values were compared across categories. A *p*‐value < .05 determined statistical significance.

Subsequently, stepwise multiple linear regression analyses were performed to identify factors independently associated with child irritability. An initial multivariable model was constructed including the family environmental variables identified in the exploratory analyses. A second model was then fitted including child sex and ADHD symptom severity (SNAP‐IV inattention and hyperactivity/impulsivity scores) as covariates to assess whether the observed associations persisted after adjustment.

We adjusted the *p*‐values for multiple comparisons using false discovery rate correction (Benjamini & Hochberg, 1995). We compared the outcome and all variables of the final multivariable model between participants with complete data and those with missing data using appropriate statistical tests.

## RESULTS

### Individual analysis of independent variables

There was no significant association between ARI scores and age or socioeconomic status. A positive correlation was found between ARI and inattention (*r* = .290, *p* < .001) and hyperactivity/impulsivity symptoms (*r* = .240, *p* = .006) (see Table 3). Additionally, ARI scores were significantly higher in boys than in girls (*W* = 15,310, *p* < .001).

**TABLE 3:** Spearman's correlation between ARI scores and quantitative independent variables.

Variable	Spearman's *ρ*	*p*‐value
Age	−.009	.918
Family income	−.160	.070
SNAP‐IV inattention	.290	<.001
SNAP‐IV hyperactivity‐impulsivity	.240	.006
Parenting		
PPS	−.391	<.001
PSOC	−.327	<.001
PS	.249	.004
Family environment		
CHAOS	.022	.803
Maternal psychopathology		
ASRS	.284	.001
ATQ‐e	−.153	.084
ATQ‐ec	−.254	.003
ATQ‐na	.243	.005
ATQ‐os	.019	.823
BDI‐II	.298	<.001
WAIS‐III	−.162	.066

Abbreviations: *ρ*, Spearman's correlation coefficient; ARI, Affective Reactivity Index; ASRS, Adult Self‐Report Scale; ATQ‐e, Adult Temperament Questionnaire – extraversion; ATQ‐ec, Adult Temperament Questionnaire – effortful control; ATQ‐na, Adult Temperament Questionnaire – negative affect; ATQ‐os, Adult Temperament Questionnaire – orienting sensitivity; BDI‐II, Beck Depression Inventory II; CHAOS, Confusion, Hubbub, and Order Scale; PPS, Parent Practices Scale; PS, Parent Scale; PSOC, Parenting Sense of Competence Scale; SNAP‐IV, Swanson, Nolan, and Pelham‐IV scale; WAIS‐III, Wechsler Adult Intelligence Scale.

Among the parenting‐related measures, a significant negative correlation was identified between ARI and the PPS scale (*r* = −.391, *p* < .001) as well as the PSOC (*r* = −.327, *p* < .001). Conversely, a significant positive correlation was found between ARI and the PS (*r* = .249, *p* = .004) (see Table 3).

Analysis of the PFMSS revealed variations in ARI scores based on different maternal speech patterns. The Wilcoxon and Kruskal–Wallis tests indicated significant differences between the medians of ARI and the response options in the following domains of the PFMSS: high EE (*W* = 2766, *p* < .001), relationship (*χ* ^2^ = 17.018, *p* < .001), and a higher frequency of negative comments compared to positive comments (*W* = 2669, *p* < .001), as shown in Table 4. Participants whose mothers exhibited low EE in the PFMSS had a lower median ARI than those with high EE. In the relationship domain, the post hoc test reveals significant differences between the groups with negative and neutral relationships (*p* = .038) and between those with negative and positive relationships (*p* < .001). The median ARI was higher among participants whose mothers had a higher frequency of negative over positive comments. However, no significant differences in ARI medians were found concerning the initial statement or the presence of warmth as measured by the PFMSS.

**TABLE 4:** Association of ARI with the PFMSS domains.

PFMSS domains	Classification	*n*	Median	Kruskal–Wallis/Wilcoxon chi‐squared	df	*p*‐value
High expressed emotion	No	65	5	2766	1	<.001
	Yes	63	7			
Relationship	Negative	48	7.5	17.67	2	<.001
	Neutral	39	6			
	Positive	41	4			
Negative comments > positive comments	No	43	3	2669	1	<.001
	Yes	85	7			
Initial statement	Negative	32	7	3.555	2	.169
	Neutral	51	6			
	Positive	45	6			
Warmth	Low	30	7	2.912	2	.233
	Moderate	45	5			
	High	53	6			

Abbreviations: ARI, Affective Reactivity Index; PFMSS, Preschool Five‐Minute Speech Sample.

Regarding the family environment, no significant correlation was observed between ARI scores and CHAOS (*r* = .022, *p* = .803). In relation to maternal psychopathology, a significant positive correlation was found between ARI and both the BDI‐II (*r* = .298, *p* < .001) and the ASRS (*r* = .284, *p* = .001). Significant associations were also identified between ARI scores and the ATQ dimensions of negative affect (ATQ‐na) (*r* = .243, *p* = .005) and effortful control (ATQ‐ec) (*r* = −.254, *p* = .003). No significant correlations were observed with the ATQ dimensions of extraversion (ATQ‐e) (*r* = −.153, *p* = .084) and orienting sensitivity (ATQ‐os) (*r* = .019, *p* = .823), or with the WAIS‐III (*r* = −.162, *p* = .066) (see Table 3).

### Multivariate model

The independent variables with statistically significant associations with the ARI were included in the stepwise multiple linear regression model. These variables were: PFMSS relationship domain, PFMSS higher frequency of negative comments compared to positive comments domain, PFMSS high EE domain, PPS, PSOC, PS, BDI‐II, ASRS, ATQ‐na, and ATQ‐ec. The second model additionally included child sex and hyperactivity/impulsivity symptoms as covariates.

The factors that were significantly associated with child irritability included two parenting‐related variables—the PFMSS higher frequency of negative comments compared to positive comments, and positive parenting practices; and one related to maternal psychopathology—the maternal ADHD symptoms, as measured by the ASRS (see Table 5). Child sex and hyperactivity/impulsivity symptoms were also retained in the final model, but family environmental factors remained independently associated with irritability. The coefficients of the full multiple linear regression model (prior to stepwise selection) are provided in Table S1.

**TABLE 5:** Multivariate model.

Variable	Estimate	Standard error	*p*‐value	BH‐adjusted *p‐*value
ASRS	0.043	0.020	.031	.030
CN > CP	1.579	0.490	.002	.004
PPS	−0.049	0.013	<.001	<.001
SNAP‐IV hyperactivity/impulsivity	1.119	0.412	.008	.009
Child sex	−1.712	0.607	.006	.009

*Note*: *p*‐values were adjusted using the Benjamini–Hochberg procedure. No statistically significant differences were observed after correction.

Abbreviations: ASRS, Adult Self‐Report Scale; BH, Benjamini–Hochberg procedure; CN > CP, negative comments > positive comments; PPS, Parent Practices Scale; SNAP‐IV, Swanson, Nolan, and Pelham‐IV scale.

No statistically significant differences were observed between participants with complete and incomplete data on the outcome or any variable included in the final multivariable model after correction for multiple comparisons (see Table S2).

## DISCUSSION

In this study, we included 128 preschool children with ADHD and investigated the relationship between a broad range of family‐related variables and child irritability. In multivariate models, less positive parenting practices and a higher frequency of negative over positive comments were the most salient factors associated with irritability in these children, alongside maternal ADHD symptoms. These findings remained significant even after adjustment for child sex and the severity of child ADHD symptoms, indicating that family environmental factors made a unique contribution to the model, beyond these covariates.

The association of child irritability with a higher frequency of negative comments in the PFMSS is consistent with longitudinal studies that have highlighted that dysfunctional parenting practices including verbal aggression and negative feeling expression are predictors of child's irritability (Derella et al., 2020; Oliver, 2015). This pattern may reflect a more critical parental perception of the child, and, given that individuals with ADHD tend to be more sensitive to perceived social rejection or criticism (Bondü & Esser, 2015), such dynamics may exacerbate emotional dysregulation. Previous research has shown robust associations between parental criticism and externalizing symptoms in childhood (Musser et al., 2016; Pauli‐Pott et al., 2021; Psychogiou et al., 2007).

Conversely, positive parenting practices emerged as a protective factor, concurring with prior evidence suggesting that supportive parenting behaviors, including emotional validation and responsiveness, are related to improved emotion regulation and encourage adaptive coping in preschool‐aged children (Li et al., 2024; Mancini et al., 2023). Our results underscore the central role of parenting in shaping emotional reactivity during early childhood, particularly in children already vulnerable due to neurodevelopmental difficulties.

Regarding maternal characteristics, higher maternal ADHD symptoms were significantly associated with greater children's irritability. ADHD is highly heritable (Faraone & Larsson, 2019) and a high prevalence of ADHD among parents of children with ADHD has been well‐documented (Efron et al., 2018; Starck et al., 2016). Thus, it is possible that children inherit a continuum of cognitive and emotional symptoms, including irritability. Nevertheless, previous literature has reported a positive correlation between ADHD symptoms in mothers and negative parenting practices, as well as lower involvement, reduced positive parenting, and higher levels of inconsistent discipline (Chronis‐Tuscano et al., 2008). Additionally, these mothers may face challenges in their own emotional regulation, further contributing to an environment more prone to stress, which may affect their children (Efron et al., 2018).

In relation to the child's ADHD symptoms, our multivariable model indicated that higher levels of hyperactive–impulsive symptoms are related to increased irritability. This observation draws attention to the study of Galera et al. (2021), who showed that children with higher levels of both ADHD symptoms and irritability tend to experience even worse outcomes in the long term. Irritability and ADHD may share various neuropsychological, neural, genetic, and environmental factors, which could help explain this link (Brotman et al., 2017). No significant associations were observed with age or socioeconomic status. Boys exhibited greater irritability than girls, consistent with the literature that shows a higher prevalence of clinically significant early childhood irritability in boys (Wiggins et al., 2023). Their overrepresentation in our sample likely reflects patterns in clinical referral for ADHD, in which boys are more frequently brought for evaluation due to more overt externalizing symptoms (Wolraich et al., 2019). It is worth noting, however, that family environmental factors remained independently associated with child irritability after including sex and ADHD symptoms in the model, demonstrating that this relationship cannot be explained solely by child ADHD symptom severity and sex.

Despite its strong association with ADHD in early childhood and its substantial long‐term functional impact, the evidence for effective treatment interventions of irritability in preschool children with ADHD remains limited. Although pharmacological treatment is often used in clinical practice, in the MAPPA study, behavioral parent training was more effective than pharmacotherapy in reducing irritability in preschoolers with ADHD (Sugaya, Salum, et al., 2022). Also, the Parent–Child Interaction Therapy with Emotion Coaching (PCIT‐Eco; Chronis‐Tuscano et al., 2016) and Parenting Your Hyperactive Preschooler (Herbert et al., 2013) are examples of evidence‐based programs that demonstrate benefits for enhancing parenting practices and improving emotion regulation and behavior in preschool children with ADHD. In brief, parent training programs teach caregivers to understand their child's maladaptive behaviors, provide clear and effective instructions, reinforce positive behaviors, and apply consistent consequences for negative ones, thereby positively impacting the child's self‐regulation process (van der Oord & Tripp, 2020).

Considering the importance of family‐focused psychosocial interventions in these cases, our study addresses a relevant gap by clarifying which specific family environmental factors should be prioritized in assessment and intervention planning. To our knowledge, this is the first study to simultaneously examine multiple components of the family environment in order to identify which factors are most strongly associated with irritability in preschool children with ADHD. We found that a focus on parenting practices and maternal psychopathology may be crucial for managing irritability in this population, highlighting the relevance of interventions that target reduction in critical and negative parental comments, increase in positive and contingent feedback, and promotion of emotionally validating responses to children's distress. Our findings also draw attention to the need to screen mothers of preschool‐aged children with ADHD for maternal ADHD symptoms, as treating this condition may be particularly beneficial in reducing irritability and enhancing child outcomes.

The results should be considered in the context of methodological limitations. The study relied on a single method for assessing irritability and family environmental factors, based on a single informant. This approach is less accurate than using multiple informants (e.g., teachers) and methods (e.g., clinical evaluation). However, data collection was conducted by professionals specialized in the field, with experience working with children, at a single research center, contributing to assessment consistency. Replication in other samples is warranted. The findings may not generalize to children from different socioeconomic or cultural backgrounds. Additionally, the overrepresentation of boys in the sample restricts the generalizability of the results to preschool girls with ADHD, pointing to the value of future studies with more balanced or sex‐stratified samples, as emphasized in recent calls within the field to increase the visibility of females in ADHD research (Becker, 2025). Another limitation was the focus on mothers in the PFMSS, as growing evidence suggests that paternal involvement is also central to child development (Amodia‐Bidakowska et al., 2020; Sarkadi et al., 2008). Moreover, the coding of this instrument by a single trained rater precluded the assessment of inter‐rater reliability. Future studies using a longitudinal design are needed to establish causal relationships between the family environmental variables and child's irritability, given the likely bidirectionality of these associations. Additionally, research should continue exploring parent‐child dynamics to better understand the potential family contributions to this emotional state. Although we included a measure of maternal temperament, future work would also benefit from directly assessing maternal irritability or emotion dysregulation, as these may play a critical role in child irritability. Despite these limitations, the results already underscore the importance of interventions tailored to the needs of both patients and their families.

## CONCLUSION

Our findings showed that parenting and maternal psychopathology were significantly related to child irritability, with the higher frequency of negative over positive comments in the PFMSS, less positive parenting practices and maternal ADHD symptoms emerging as specific factors. This study has valuable implications for the understanding of these key family environmental contributors to irritability in preschoolers with ADHD, suggesting potential targets for family‐based early intervention. Enhancing positive parenting practices, reducing criticism and addressing maternal psychopathology could be crucial in mitigating children's irritability and its long‐term consequences.

## AUTHOR CONTRIBUTIONS

**Analin Ono Baraniuk**: Conceptualization; methodology; data curation; writing—original draft; writing—review and editing; visualization. **Giovanni Abrahão Salum**: Writing—review and editing. **David Daley**: Writing—review and editing. **Erika Mendonça de Morais**: Investigation. **Filipe Jaeger Zabala**: Formal analysis. **Luiz Eduardo de Souza**: Formal analysis. **Guilherme V. Polanczyk**: Conceptualization; methodology; writing—review and editing; supervision. **Luisa Shiguemi Sugaya**: Conceptualization; supervision; writing—review and editing; methodology.

## CONFLICT OF INTEREST STATEMENT

G. V. P. has served as a speaker and/or consultant to Abbott, Ache, Adium, Apsen, EMS, Libbs, Medice, Takeda, and receives authorship royalties from Manole Editors. The remaining authors have declared that they have no competing or potential conflicts of interest.

## ETHICAL CONSIDERATIONS

Parental consent was obtained from all participants. The study was approved by the Research Ethics Committee (Comissão de Ética para Análise de Projetos de Pesquisa – CAPPesq) of Hospital das Clínicas, University of São Paulo, Brazil (Approval number 6.752.244, February 9, 2024).

## Supporting information

Tables S1 and S2

## Data Availability

The data that support the findings of this study are available from the corresponding author upon reasonable request.
