# Supplementary material for: Irritability in preschool children with attention‐deficit/hyperactivity disorder: Analysis of family environmental factors
Source: JCPP Adv. 2026 Apr 8:e70120. Online ahead of print. doi: 10.1002/jcv2.70120 (PMC13339585; doi:10.1002/jcv2.70120)
Supplement: Supplementary file 1 — Tables S1 and S2 [file JCV2-9999-e70120-s001.docx]

**Irritability in preschool children with attention-deficit/hyperactivity disorder: analysis of family environmental factors**

**Supporting Information**

**Table S1**

*Coefficients of the Full Multiple Linear Regression Model (Prior to Stepwise Selection)*

| **Variable** | **Estimate** | **Standard error** | ***P*-value** | **BH-adjusted**  ***P*-value** |
| --- | --- | --- | --- | --- |
| **ASRS** | 0.027 | 0.028 | 0.339 | 0.584 |
| **ATQ-ec** | −0.039 | 0.141 | 0.781 | 0.841 |
| **ATQ-na** | 0.014 | 0.084 | 0.871 | 0.870 |
| **BDI-II** | 0.025 | 0.029 | 0.377 | 0.584 |
| **CN > CP**  **(PFMSS)** | 1.450 | 0.734 | 0.051 | 0.236 |
| **High expressed emotion (PFMSS)** | −0.883 | 0.880 | 0.318 | 0.584 |
| **PS** | 0.166 | 0.428 | 0.700 | 0.841 |
| **PPS** | −0.038 | 0.017 | 0.022 | 0.155 |
| **PSOC** | 0.011 | 0.036 | 0.772 | 0.841 |
| **Neutral relationship (PFMSS)** | −0.612 | 0.748 | 0.415 | 0.584 |
| **Positive relationship (PFMSS)** | −1.371 | 0.959 | 0.156 | 0.435 |
| **Child sex** | −1.781 | 0.633 | 0.006 | 0.081 |
| **SNAP-IV hyperactivity/impulsivity** | 0.784 | 0.525 | 0.138 | 0.435 |
| **SNAP-IV inattention** | 0.445 | 0.547 | 0.418 | 0.584 |

*Note*. ASRS = Adult Self-Report Scale; ATQ-ec = Adult Temperament Questionnaire - effortful control; ATQ-na = Adult Temperament Questionnaire - negative affect; BDI-II = Beck Depression Inventory II; BH = Benjamini–Hochberg procedure; CN > CP = Negative comments > positive comments; PFMSS = Preschool Five-Minute Speech Sample; PS = Parent Scale; PSOC = Parenting Sense of Competence Scale; PPS = Parent Practices Scale; SNAP-IV = Swanson, Nolan, and Pelham-IV scale.

*P*-values were adjusted using the Benjamini–Hochberg procedure. The loss of significance in the saturated model likely reflects overlapping variance among the variables, whereas the optimized model obtained through a stepwise procedure highlights the most relevant and independent environmental correlates of irritability, which remained statistically significant after correction for multiple comparisons.

**Table S2**

*Group Comparisons Between Participants With and Without Missing Data in the Final Multivariable Model (Benjamini–Hochberg Correction)*

| **Variable** | **Test used** | **Raw *P*-value** | **BH-adjusted *P*-value** |
| --- | --- | --- | --- |
| **ARI** | Wilcoxon rank-sum | 0.644 | - |
| **ASRS** | Wilcoxon rank-sum | 0.219 | 0.412 |
| **CN > CP** | Fisher’s exact test | 0.247 | 0.412 |
| **PPS** | Wilcoxon rank-sum | 0.642 | 0.803 |
| **SNAP-IV hyperactivity/impulsivity** | Wilcoxon rank-sum | 0.044 | 0.223 |
| **Child sex** | Fisher’s exact test | 1.000 | 1.000 |

*Note.* ASRS = Adult Self-Report Scale; BH = Benjamini–Hochberg procedure; CN > CP = Negative comments > positive comments; PPS = Parent Practices Scale; SNAP-IV = Swanson, Nolan, and Pelham-IV scale.

*Note.* Wilcoxon rank-sum and Fisher’s exact test were used as appropriate. *P*-values were adjusted using the Benjamini–Hochberg procedure. No statistically significant differences were observed after correction.
